# *RFC1* repeat expansions in downbeat nystagmus syndromes: frequency and phenotypic profile

**DOI:** 10.1007/s00415-024-12229-z

**Published:** 2024-02-21

**Authors:** David Pellerin, Felix Heindl, Andreas Traschütz, Dan Rujescu, Annette M. Hartmann, Bernard Brais, Henry Houlden, Claudia Dufke, Olaf Riess, Tobias Haack, Michael Strupp, Matthis Synofzik

**Affiliations:** 1 Department of Neurology and Neurosurgery, Montreal Neurological Hospital and Institute, McGill University, Montreal, QC Canada; 2 Department of Neuromuscular Diseases, UCL Queen Square Institute of Neurology and The National Hospital for Neurology and Neurosurgery, University College London, London, UK; 3 Department of Neurology and German Center for Vertigo and Balance Disorders, University Hospital, Ludwig-Maximilians University, Munich, Germany; 4 Division Translational Genomics of Neurodegenerative Diseases, Hertie-Institute for Clinical Brain Research and Center of Neurology, University of Tübingen, Tübingen, Germany; 5 German Center for Neurodegenerative Diseases (DZNE), Tübingen, Germany; 6 Department of Psychiatry and Psychotherapy, Comprehensive Center for Clinical Neurosciences and Mental Health (C3NMH), Medical University of Vienna, Vienna, Austria; 7 Department of Human Genetics, McGill University, Montreal, QC Canada; 8 Centre de Réadaptation Lucie-Bruneau, Montreal, QC Canada; 9 Institute of Medical Genetics and Applied Genomics, University of Tübingen, Tübingen, Germany

**Keywords:** *FGF14*, SCA27B, GAA-FGF14 ataxia, CANVAS, Cerebellar ataxia, Bilateral vestibulopathy

## Abstract

**Objectives:**

The cause of downbeat nystagmus (DBN) remains unknown in a substantial number of patients (“idiopathic”), although intronic GAA expansions in *FGF14* have recently been shown to account for almost 50% of yet idiopathic cases. Here, we hypothesized that biallelic *RFC1* expansions may also represent a recurrent cause of DBN syndrome.

**Methods:**

We genotyped the *RFC1* repeat and performed in-depth phenotyping in 203 patients with DBN, including 65 patients with idiopathic DBN, 102 patients carrying an *FGF14* GAA expansion, and 36 patients with presumed secondary DBN.

**Results:**

Biallelic *RFC1* AAGGG expansions were identified in 15/65 patients with idiopathic DBN (23%). None of the 102 GAA-*FGF14*-positive patients, but 2/36 (6%) of patients with presumed secondary DBN carried biallelic *RFC1* expansions. The DBN syndrome in *RFC1*-positive patients was characterized by additional cerebellar impairment in 100% (15/15), bilateral vestibulopathy (BVP) in 100% (15/15), and polyneuropathy in 80% (12/15) of cases. Compared to GAA-*FGF14*-positive and genetically unexplained patients, *RFC1*-positive patients had significantly more frequent neuropathic features on examination and BVP. Furthermore, vestibular function, as measured by the video head impulse test, was significantly more impaired in *RFC1*-positive patients.

**Discussion:**

Biallelic *RFC1* expansions are a common monogenic cause of DBN syndrome.

## Introduction

Until recently, the cause of downbeat nystagmus (DBN) has remained unknown (“idiopathic”) in approximately 30% of cases [1]. However, intronic *FGF14* (GAA)_≥250_ repeat expansions, known to cause spinocerebellar ataxia 27B/GAA-*FGF14* disease [2, 3], were lately shown to account for almost 50% of previously unexplained DBN cases [4], suggesting that monogenic causes may be a recurrent cause of what has so far been considered “idiopathic” DBN.

In particular, biallelic *RFC1* repeat expansions may represent a common cause of “idiopathic” DBN syndrome given the anecdotal reports of DBN in *RFC1*-related disorder [5–9]. To test this hypothesis, we studied the frequency of *RFC1* repeat expansions in a cohort of patients with “idiopathic” DBN, characterized the phenotypic profile of the *RFC1-*related DBN syndrome, and compared it to that of the GAA*-FGF14*-related DBN syndrome.

## Methods

**Fig. 1:**
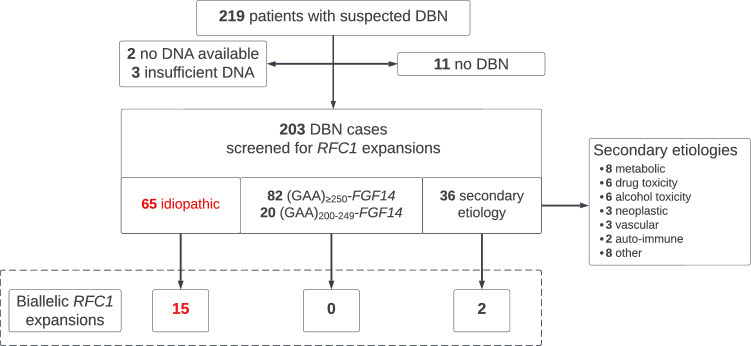
Study flowchart of the recruitment of patients with DBN. *DBN* downbeat nystagmus

We studied a series of 219 patients with suspected DBN of unknown etiology referred to the Department of Neurology or the German Center for Vertigo and Balance Disorders at the LMU Hospital in Munich, Germany, between 2012 and 2020. Patients underwent comprehensive etiologic evaluation of DBN syndrome and in-depth phenotyping as described previously [4]. Patients were excluded from the study if no or insufficient DNA was available for genetic screening (*n* = 5) or if DBN was not objectified on examination (*n* = 11) (Fig. 1). Of the remaining 203 patients with DBN, a presumed secondary cause of DBN—either acquired or genetic, but excluding GAA-*FGF14* disease—had previously been identified in 36 patients during clinical and paraclinical evaluation, an *FGF14* (GAA)_≥250_ allele in 82 patients, and an *FGF14* (GAA)_200–249_ allele in 20 patients, yielding 65 patients with “idiopathic” DBN (Fig. 1). Patients carrying an *FGF14* (GAA)_≥250_ allele and a (GAA)_200–249_ allele were analyzed together due to recent evidence suggesting that (GAA)_200–249_ alleles may be associated with DBN, given their significant enrichment in patients with DBN and that the phenotype of (GAA)_200–249_-*FGF14* patients closely mirrored that of (GAA)_≥250_-*FGF14* patients [4]. All 203 patients with DBN were screened for *RFC1* AAGGG expansions as described previously [10]. Patients with a presumed secondary cause of DBN and GAA-*FGF14*-related DBN were not excluded from *RFC1* screening to explore the possibility of co-occurring diseases. Two patients with a presumed secondary cause of DBN (chronic alcohol use) who were found to carry biallelic *RFC1* repeat expansions were not included in the phenotypic analysis of the *RFC1*-related DBN syndrome cohort given the difficulty in determining the relative phenotypic contributions of chronic alcohol use and *RFC1* repeat expansions.

Deep phenotyping was performed by reassessing medical records using a standardized data sheet. Bilateral vestibulopathy (BVP) was diagnosed as per the consensus criteria of the Bárány Society requiring the documentation of bilaterally reduced or absent angular vestibular ocular reflex (VOR) function by caloric stimulation, video head impulse test (vHIT), or rotatory chair [11]. Polyneuropathy was diagnosed on nerve conduction studies (NCS), excluding focal entrapment neuropathies, or clinically defined by the combination of significantly decreased vibration sense at the ankles (≤ 3/8 on the Rydel–Seiffer scale) and decreased ankle reflexes [12].

This study was approved by the ethics committee of the LMU Munich and we obtained written informed consent from all the participants.

## Results

### Frequency of biallelic *RFC1* expansions

**Fig. 2:**
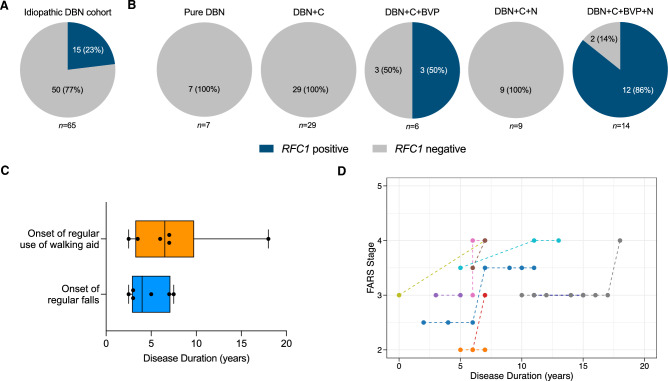
Frequency of *RFC1* repeat expansions in DBN syndromes and progression of functional disability in the *RFC1*-related DBN syndrome. **A** Percentage of patients carrying biallelic *RFC1* AAGGG repeat expansions in a cohort of 65 patients with idiopathic downbeat nystagmus (DBN). **B** Percentage of patients carrying biallelic *RFC1* AAGGG repeat expansions in the phenotypic subgroups with (1) pure DBN, (2) DBN plus cerebellar impairment (DBN + C), (3) DBN plus cerebellar impairment and bilateral vestibulopathy (DBN + C + BVP), (4) DBN plus cerebellar impairment and polyneuropathy (DBN + C + N), and (5) DBN plus cerebellar impairment, BVP, and polyneuropathy (DBN + C + BVP + N). No patient with DBN plus isolated BVP or isolated neuropathy was identified among the idiopathic DBN cohort. **C** Disease duration at time of onset of regular use of walking aid and regular falls in the *RFC1*-positive patients with DBN. **D** Longitudinal intra-individual progression of functional impairment as assessed by the FARS functional disability stage relative to disease duration (35 observations from 11 patients with DBN carrying biallelic *RFC1* repeat expansions). Observations from the same patient are connected by a dotted line. The FARS functional stage assesses disability through a 7-point ordinal scale: 0 = normal; 1 = minimal signs on examination; 2 = minimal disability; 3 = mild disability; 4 = moderate disability, requires a walker; 5 = severe disability, confined but can navigate a wheelchair; 6 = total disability

Biallelic *RFC1* AAGGG repeat expansions were identified in 15 of 65 (23%) patients with “idiopathic” DBN (Figs.1 and 2A). Moreover, a high frequency of heterozygous *RFC1* repeat expansion carriers was observed in the “idiopathic” DBN cohort (12%, 8/65 patients / 6.2%, 8/130 allele frequency; compared to 0.7–6.5% carrier frequency in the general population [13]). A total of 50 patients remained unsolved after *RFC1* screening, and will be referred to onward as “genetically unexplained”. In addition, 2 of the 36 (6%) patients with presumed secondary DBN were found to carry biallelic *RFC1* AAGGG repeat expansions, while none of the 102 (GAA)_≥200_-*FGF14*-positive patients did (Fig. 1).

### Phenotypic characterization of the *RFC1*-related DBN syndrome

The frequency of biallelic *RFC1* repeat expansions stratified by DBN subgroups was 50% (3/6) for DBN plus cerebellar impairment and BVP, and 86% (12/14) for DBN plus cerebellar impairment, BVP, and polyneuropathy (Fig. 2B). None of the patients in the other DBN subgroups carried biallelic *RFC1* repeat expansions (Fig. 2B). Table 1 presents the clinical features of the *RFC1*-positive, (GAA)_≥200_-*FGF14*-positive, and genetically unexplained DBN cohorts.

**Table 1:** Characteristics and discriminative features of the *RFC1*-related downbeat nystagmus syndrome

	*RFC1*-positive group (*n* = 15)	(GAA)_≥200_-*FGF14*-positive group (*n* = 102)	Genetically unexplained group (*n* = 50)	*RFC1*-positive vs GAA-*FGF14*-positive	*RFC1*-positive vs genetically unexplained
				*p* value	*p* value
Male sex	7 (74%)	55 (54%)	31 (62%)	–	–
Age at disease onset	63.5 (44–78)	67 (30–84)	67 (17–88)	0.080	0.262
Disease duration	7 (4–18)	6 (0–26.5)	4 (0–50)	0.070	**0.007**
Age at last examination	72 (52–91)	74.5 (40–92)	72 (21–89)	0.210	0.878
Positive family history	4/15 (27%)	35/102 (34%)	7/49 (14%)	0.771	0.268
FARS disability stage^a^	3.25 (1.5–4)	3 (1.5–5)	2 (1–4)	**0.042**	**0.003**
History of falls	8/9 (89%)	35/64 (55%)	10/23 (43%)	0.072	**0.044**
Regular use of walking aid	7/14 (50%)	19/99 (19%)	7/49 (14%)	**0.017**	**0.009**
Symptoms					
Episodic symptoms	0/14 (0%)	11/100 (11%)	18/49 (37%)	0.354	**0.006**
Postural instability	15/15 (100%)	101/101 (100%)	50/50 (100%)	1.000	1.000
Visual disturbances	9/15 (60%)	55/102 (54%)	20/50 (40%)	0.784	0.238
Fine motor impairment	3/14 (21%)	13/98 (13%)	8/49 (16%)	0.419	0.419
Speech impairment	3/15 (20%)	17/100 (17%)	8/48 (17%)	0.723	0.714
Swallowing difficulties	1/15 (7%)	7/100 (7%)	4/49 (8%)	1.000	1.000
Sensory symptoms	5/15 (33%)	13/100 (13%)	7/49 (14%)	0.058	0.132
Autonomic symptoms	3/15 (20%)	9/101 (9%)	3/48 (6%)	0.187	0.141
Clinical signs					
Impaired balance/gait	15/15 (100%)	80/98 (82%)	27/46 (59%)	0.123	**0.003**
Positive Romberg test	12/13 (92%)	48/87 (55%)	15/38 (39%)	**0.013**	**0.001**
Cerebellar ocular motor signs					
Gaze–evoked nystagmus	10/15 (67%)	68/102 (67%)	30/49 (61%)	1.000	0.769
Saccadic pursuit	15/15 (100%)	100/101 (99%)	41/49 (84%)	1.000	0.181
Dysmetric saccades	6/15 (40%)	28/99 (28%)	14/49 (29%)	0.374	0.526
Cerebellar ataxia					
Ataxia of upper limbs	8/14 (57%)	17/87 (20%)	11/38 (29%)	**0.005**	0.103
Dysdiadochokinesia	5/13 (38%)	16/87 (18%)	9/40 (22%)	0.139	0.292
Dysarthria	4/15 (27%)	14/99 (14%)	6/45 (13%)	0.252	0.250
Neuropathy					
Impaired vibration at ankle (≤ 3/8)	11/14 (79%)	17/97 (18%)	11/48 (23%)	** < 0.001**	** < 0.001**
Ankle hyporeflexia	10/14 (71%)	24/97 (25%)	17/48 (35%)	** < 0.001**	**0.030**
Pyramidal tract signs	0/14 (0%)	1/95 (1%)	1/49 (2%)	1.000	1.000
Parkinsonism	2/14 (14%)	13/97 (13%)	10/49 (20%)	1.000	1.000
MRI					
Disease duration at last MRI	6 (5–15)	4 (0–17)	2 (− 3–50)	**0.013**	**0.007**
Vermis atrophy	4/11 (36%)	9/71 (13%)	5/33 (15%)	0.068	0.195
Cerebellar hemisphere atrophy	3/11 (27%)	5/71 (7%)	4/33 (12%)	0.070	0.341
Brainstem atrophy	1/11 (9%)	0/70 (0%)	1/33 (3%)	0.136	0.442
Nerve conduction studies					
Abnormal sural SNAP	6/6 (100%)	10/20 (50%)	3/3 (100%)	0.157	1.000
Abnormal upper limb SNAP	4/4 (100%)	1/10 (10%)	0/2 (0%)	**0.005**	0.067
Abnormal CMAP (any nerve)	2/5 (40%)	7/20 (35%)	3/3 (100%)	1.000	0.196
Vestibular function evaluation—caloric stimulation, vHIT, rotatory chair					
Bilateral vestibulopathy	15/15 (100%)	11/97 (11%)	5/45 (11%)	** < 0.001**	** < 0.001**
VOR gain on vHIT in BVP—mean (± SD)	0.15 (± 0.11)	0.39 (± 0.15)	0.50 (± 0.07)	**0.004**	**0.036**
Response to 4-aminopyridine treatment					
Clinician-reported response	1/6 (17%)	33/41 (80%)	4/9 (44%)	**0.004**	0.580
Patient-reported response	1/7 (14%)	32/54 (59%)	1/11 (9%)	**0.041**	1.000

Unless specified, data are reported as frequencies (percentages) for qualitative variables and median (range) for quantitative variables. Differences between groups were assessed with the non-parametric Mann–Whitney *U* test for continuous variables and the Fisher’s exact test for categorical variables. Bold values indicate statistically significant *p* values. Data on age at onset were missing for three patients in the *RFC1*-positive group, eleven patients in the (GAA)_≥200_-*FGF14*-positive group, and five patients in the genetically unexplained group

*BVP* Bilateral vestibulopathy, *CMAP* Compound motor action potential, *FARS* Friedreich Ataxia Rating Scale, *SNAP* Sensory nerve action potential, *vHIT* Video head impulse test, *VOR* Vestibulo-ocular reflex

^a^Last available FARS disability stage measured off 4-aminopyridine

DBN occurred with cerebellar impairment in all 15 *RFC1*-positive patients, which was limited to the ocular motor system with typical cerebellar ocular motor signs (i.e., saccadic pursuit, dysmetric saccades, gaze-evoked nystagmus) in 5 patients (33%). Additional cerebellar ocular motor signs were observed in all *RFC1*-positive patients. Brain MRI showed global cerebellar atrophy in 27% (3/11) and isolated vermis atrophy in 9% (1/11) of patients. BVP was documented in all *RFC1*-positive patients by vHIT (*n* = 10) or caloric stimulation (*n* = 5). Polyneuropathy was identified in 12 of 15 (80%) *RFC1*-positive patients, and was diagnosed on NCS in six patients and clinically in six patients. Three patients had no evidence of neuropathic features on examination, though NCS were not available for these patients. The presence of chronic cough could not be reliably extracted from medical records, although it was documented in two patients in whom it developed more than 10 years before the onset of gait impairment.

### Progression of functional disability in the *RFC1*-related DBN syndrome

A substantial proportion of *RFC1*-positive patients experienced regular falls (89%, 8/9), some of them as early as 2.5 years after disease onset (median disease duration at onset of regular falls, 4 years; range, 2.5–7.5). Furthermore, walking aids were used by 50% of patients (7/14) after a median disease duration of 6.5 years (range, 2.5–18) (Fig. 2C). At time of last examination, the median Friedreich Ataxia Rating Scale (FARS) functional stage was 3.25 (range, 1.5–4), indicating a mild-to-moderate disability (Fig. 2D).

### Discriminative features of the *RFC1*-related DBN syndrome

Compared to (GAA)_≥200_-*FGF14*-positive and genetically unexplained patients with DBN, *RFC1*-positive patients with DBN appeared more functionally impaired, as assessed by the FARS functional stage, history of regular falls and use of walking aids, and gait impairment on examination (Table 1). However, the *RFC1*-positive DBN group also had a significantly longer disease duration compared to the genetically unexplained group (median, 7 vs 4 years; *p* = 0.007) and a trend toward longer disease duration compared to the (GAA)_≥200_-*FGF14*-positive group (median, 7 vs 6 years; *p* = 0.070), which may account in part for the higher degree of functional impairment in the *RFC1*-positive group. Neuropathic features and proprioceptive dysfunction on examination were significantly more common in the *RFC1*-positive group (Table 1), in keeping with early and preferential involvement of dorsal root ganglia in that disease [14]. Vestibular impairment was also significantly more common and severe, as measured by VOR gains on vHIT, in *RFC1*-positive patients (*n* = 9) compared to (GAA)_≥200_-*FGF14*-positive (*n* = 9) and genetically unexplained patients (*n* = 2) (Table 1).

## Discussion

This study showed that biallelic *RFC1* AAGGG repeat expansions are a common monogenic cause of DBN syndrome, accounting for 23% of previously “idiopathic” DBN cases. Given this high frequency, genetic testing for *RFC1* repeat expansions may now become part of the diagnostic workup of patients with “idiopathic” DBN. Of note, since biallelic *RFC1* repeat expansions were also identified in 6% of patients who had a presumed secondary cause of DBN, genetic testing might need to be extended to this population as well—especially in the presence of other cerebellar signs, vestibular hypofunction, and/or polyneuropathy—given the implications for clinical management and eligibility for future clinical trials.

Our findings provide a deeper phenotypic characterization of the *RFC1*-related DBN syndrome by showing that they present along a continuum of involvement of the cerebellar, sensory, and vestibular systems. This confirms and extends previous notions of widespread neurodegeneration occurring in *RFC1*-related disorder [15]. Accordingly, no patient with pure DBN or DBN plus cerebellar impairment (without BVP and/or polyneuropathy) was found to carry biallelic *RFC1* repeat expansions, strengthening the observation that *RFC1*-related disorder is unlikely in presence of isolated cerebellar ataxia without sensory neuropathy [16]. The multisystemic involvement in the *RFC1*-positive DBN syndrome was further reflected by the significantly more common neuropathic features and proprioceptive dysfunction on examination as well as vestibular impairment—which was comparatively more severe—in this group compared to the (GAA)_≥200_-*FGF14*-positive and genetically unexplained groups. These phenotypic findings might help to raise clinical suspicion for *RFC1*-related disease over other monogenic causes of DBN, such as GAA-*FGF14* disease [4].

Our study also provides preliminary insights into the natural evolution of the *RFC1*-related DBN syndrome. A significant proportion of patients experienced regular falls and needed walking aids relatively early in the disease course, which is of importance for clinical management given the relevance for everyday living and as potentially highly meaningful outcomes in future treatment trials [17]. However, it remains to be established in larger, prospective cohort series if functional impairment progresses more rapidly in the *RFC1*-related DBN syndrome compared to the GAA-*FGF14*-related DBN syndrome, which would be in line with the higher degree of underlying multisystemic neurodegeneration in *RFC1*-related disease [15, 18].

Our study has several limitations. First, it is a single-centre retrospective study, which limited our ability to assess the evolution of multisystemic damage in the *RFC1*-positive DBN syndrome. Second, our study provides a conservative estimate of the real frequency of *RFC1*-related disorder in “idiopathic” DBN as it only screened for pathogenic AAGGG repeat motifs and not for truncating variants and other non-reference pathogenic motifs that have recently been shown to cause *RFC1*-related disorder [7, 19]. The elevated frequency of heterozygous *RFC1* repeat expansion carriers in our cohort (12%) raises the possibility that some of these patients may carry a novel variant in *trans* with the AAGGG expansion. Third, we were unable to objectify the presence of polyneuropathy—a universal feature of *RFC1*-related disorder [14, 15]—in all *RFC1*-positive patients as only 40% underwent NCS.

In conclusion, we showed that biallelic *RFC1* AAGGG repeat expansions are a recurrent monogenic cause of DBN syndrome.

## Data Availability

Individual deidentified patient data may be shared at the request of any qualified investigator upon reasonable request.
